# Identification of key genes involved in the phenotypic alterations of *res* (*restored cell structure by salinity*) tomato mutant and its recovery induced by salt stress through transcriptomic analysis

**DOI:** 10.1186/s12870-018-1436-9

**Published:** 2018-10-01

**Authors:** Irene Albaladejo, Isabel Egea, Belen Morales, Francisco B. Flores, Carmen Capel, Rafael Lozano, Maria C. Bolarin

**Affiliations:** 1 Departamento de Biología del Estrés y Patología Vegetal, Centro de Edafología y Biología Aplicada del Segura, CSIC, Campus Universitario Espinardo, 30100 Murcia, Spain; 2 Centro de Investigación en Biotecnología Agroalimentaria (BITAL), Universidad de Almería, 04120 Almería, Spain

**Keywords:** *Solanum lycopersicum*, *res* mutant, Microarrays, Growth-defence tradeoff, Salt stress

## Abstract

**Background:**

The *res* (*re*stored cell structure by *s*alinity) mutant, recently identified as the first tomato mutant accumulating jasmonate in roots under non-stressful conditions, exhibits a remarkable growth inhibition and morphological alterations in roots and leaves, which are suppressed when the mutant plants are exposed to salinity. In order to understand the molecular basis of the phenotype recovery induced by salt stress in the *res* mutant, we carried out a comparative transcriptomic analysis in roots and leaves of wild-type and *res* plants in absence of stress (control) and when the phenotypic recovery of *res* mutant began to be observed upon salt stress (5 days of 200 mM NaCl).

**Results:**

The number of differentially expressed genes was three times greater in roots than in leaves of *res* vs WT plants grown in control, and included the down-regulation of growth-promoting genes and the up-regulation of genes involved in Ca^2+^ signalling, transcription factors and others related to stress responses. However, these expression differences were attenuated under salt stress, coinciding with the phenotypic normalisation of the mutant. Contrarily to the attenuated response observed in roots, an enhanced response was found in leaves under salt stress. This included drastic expression changes in several circadian clock genes, such as *GIGANTEA1*, which was down-regulated in *res* vs WT plants. Moreover, the higher photosynthetic efficiency of *res* leaves under salt stress was accompanied by specific salt-upregulation of the genes *RUBISCO ACTIVASE1* and *ALTERNATIVE OXIDASE1A*. Very few genes were found to be differentially expressed in both tissues (root and leaf) and conditions (control and salt), but this group included *SlWRKY39* and *SlMYB14* transcription factors, as well as genes related to protein homeostasis, especially protease inhibitors such as *METALLOCARBOXYPEPTIDASE INHIBITOR*, which also seem to play a role in the phenotype recovery and salt tolerance of *res* mutant.

**Conclusions:**

In summary, in this study we have identified genes which seem to have a prominent role in salt tolerance. Moreover, we think this work could contribute to future breeding of tomato crops with increased stress tolerance.

**Electronic supplementary material:**

The online version of this article (10.1186/s12870-018-1436-9) contains supplementary material, which is available to authorized users.

## Background

Agriculture is probably facing its biggest challenge in human history due to world climate change, as the average global earth surface temperature is significantly rising, drastically affecting global agricultural systems, especially in arid and semi-arid areas, which represent about one-third of the planet surface [1]. In these areas, salinization is another important problem caused by the frequent use of irrigation waters that contain salts as NaCl. To face this situation, it is crucial to unravel the key components of the plant salt-tolerance network [2], as advances in the understanding of stress signalling and responses will increase our ability to improve stress resistance in crops, and thus to achieve agricultural sustainability and food security for a growing world population [3]. A generic signal transduction pathway starts with the perception of outside signals, followed by a series of intracellular reactions, including the generation of second messengers, changes in intracellular Ca^2+^ levels, the initiation of a protein phosphorylation cascade, and finally, the activation of target proteins directly involved in cellular protection or transcription factors (TFs) controlling specific sets of stress-regulated genes [2, 4, 5].

Plant hormones are central regulators of complex developmental processes and stress-adaptative signalling cascades [6], and it has been suggested that the balance between different hormones determines the appropriate response to an experienced stress [7]. Among them, jasmonate (JA) is considered a key regulator of stress responses in virtually all plant species [8, 9]. There is increasing evidence that JA mediates multiple stress responses, from biotic stresses and mechanical wounding to different abiotic stresses, including salinity [10, 11], although the activation of JA signalling severely restricts plant growth [12]. Recently, we identified the *res* tomato mutant, which in absence of stress presented remarkable morphological alterations, growth inhibition and cellular disorganization in roots and leaves, including alterations in chloroplast structure [13]. Moreover, this mutant contains high JA level and increased expression of genes involved in JA biosynthesis and signalling pathways in roots, plant organ where investigations are scarce, as most of studies on tolerance induced by JA to abiotic and biotic stresses are referred to its biosynthesis in leaves [8, 14]. Surprisingly, under salt stress the *res* mutant was able to restore a normal phenotype and cell structure, which in turn resulted in increased growth of *res* plants [13]. Moreover, *res* plants were also able to recover a normal phenotype when exposed to high temperatures and low relative humidity in the natural summer conditions of the Mediterranean area [15]. In sum, *res* mutant seems to be prepared to confront abiotic stresses and growth inhibition may represent the cost for that benefit. In this sense, the tolerance of halophytic species is generally accompanied by slow plant development, which has been attributed to the energetic cost that implies high basal levels of genes in absence of stress [16, 17].

In tomato (*Solanum lycopersicum*), considered one of the most economically important horticultural crops grown worldwide [18], and a highly important crop in agriculture of arid and semi-arid zones, studies of mutants with constitutive stress responses are very scarce, despite the analysis of these kind of mutants may be very helpful to identify key genes involved in plant adaptation and survival upon salt stress [19]. The comparative transcriptomic analysis between the tomato *res* mutant and its background genotype (cv Moneymaker) revealed a constitutive alteration of an important number of genes involved in different pathways in *res* mutant, and identified genes specifically overexpressed under salt stress and responsible of both maintaining plant growth and promoting stress tolerance in *res* mutant.

## Results

**Fig. 1:**
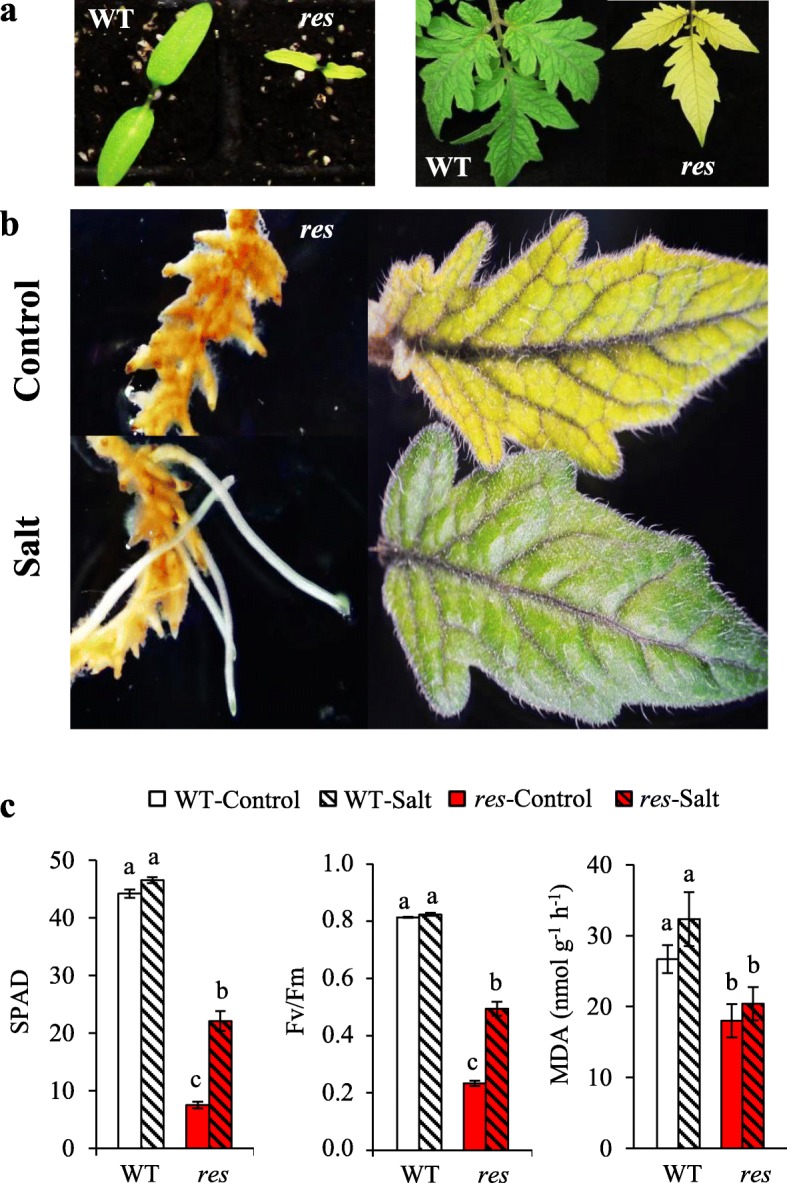
Normalisation of the *res* tomato mutant phenotype. (**a**) Comparison of WT and *res* seedlings (left) and leaves of adult plants (right) in absence of salt stress. (**b**) Phenotypic changes provoked by salt treatment (5 days at 200 mM NaCl) in roots and leaves of *res*. (**c**) Chlorophyll content (SPAD), photosynthetic efficiency (Fv/Fm) and MDA content measured in WT (white bars) and *res* (red bars) leaves under control conditions (plain coloured bars) and after 5 days of salt stress (dashed coloured bars). Values are means ± SE of three biological replicates. Different letters indicate significant differences between means by LSD test (*P < 0.05*)

The *res* mutant is chlorotic from the cotyledon stage (Fig. 1a), and maintains leaf chlorosis throughout the plant development (Additional file 1: Figure S1), but is able to restore a normal phenotype under salt stress [13]. In order to elucidate the molecular basis involved in the phenotype recovery induced by salinity in *res*, we previously selected a time of salt exposure corresponding to the middle of the phenotype recovery. The period of salt stress applied (5 days of 200 mM NaCl) was selected because the phenotypic normalisation of *res* mutant began to be observed in leaves and roots (Fig. 1b, Additional file 1: Figure S1). Moreover, at this time the chlorophyll and photosynthetic efficiency values in leaves of salt-treated *res* plants were at least twice greater than those of the *res* control leaves and around 50% compared to leaves of WT (Fig. 1c). We also observed that MDA content, which allows the estimation of oxidative damage on plants, was lower in the leaves of the mutant compared to those of WT, both in control and salt stress (Fig. 1c). On the other hand, WT plants did not show significant physiological changes after this period of salt treatment compared to non-stressful conditions, which agrees with our previous research with WT plants under these conditions [13].

### Comparative transcriptome analysis between *res* mutant and WT plants in absence of stress and after salt treatment

**Fig. 2:**
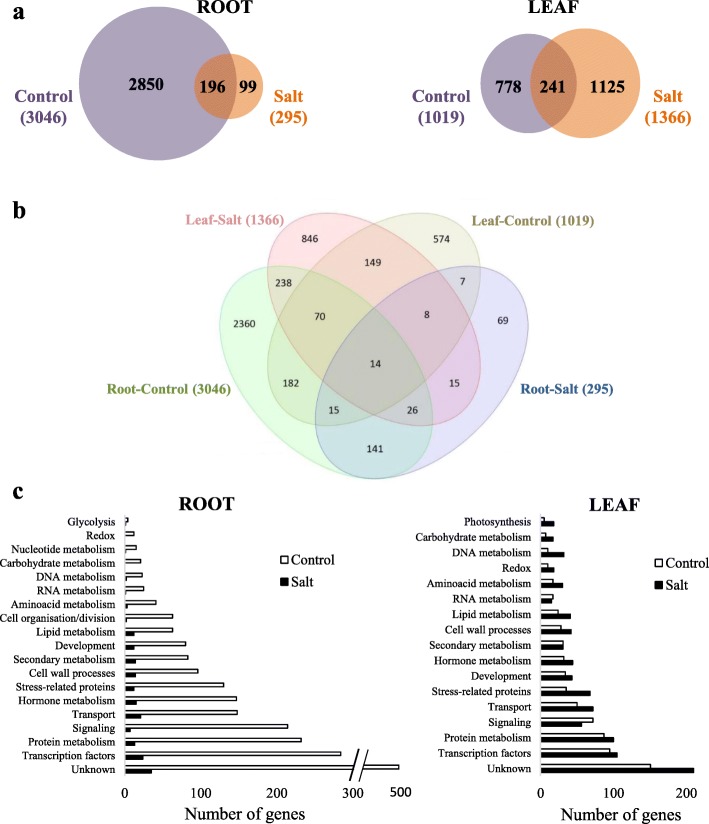
Gene expression differences between WT and *res* plants in roots and leaves. (**a**) Venn diagrams showing the number of differentially expressed genes (DEGs) comparing WT and *res* in absence of salt stress (control) and exposed to 200 mM NaCl for 5 days (Salt), as well as the overlap of genes commonly expressed in both conditions. Numbers in parentheses are the total number of DEGs in control and salt stress in both tissues. DEGs are those showing a FDR < 0.05 and a minimum fold-change value of 2.0. (**b**) Four-way Venn diagram showing the relationship between DEGs identified in each tissue and treatment. Leaf-Control and Root-Control represent DEGs in *res* vs WT in each tissue in absence of salt stress, whereas Leaf-Salt and Root-Salt include DEGs in *res* vs WT in each tissue during salt stress. (**c**) Number of DEGs classified in functional categories for each tissue using Mapman, both in control and salt stress

Regarding the transcriptomic analysis, the comparison of *res* vs WT grown in control allowed to identify constitutive gene expression differences between genotypes, whereas *res* vs WT in salt stress identified DEGs in salinity, which may be constitutive or not; in addition, each genotype was compared in salt stress vs control, in order to detect transcripts specifically altered by salinity in WT and mutant plants. A summary of the differences observed when comparing the transcriptome of WT and *res* plants in each treatment is presented in Fig. 2. DEGs between WT and *res* mutant were very high (3046 DEGs) in roots under control conditions, whereas only 295 DEGs (approximately a 10%) were detected in salt-stressed roots (Fig. 2a). However, the DEG number in leaves was slightly lower in control than in salt (1019 and 1366 DEGs, respectively), of which only 241 (a 20% approximately) were common to both conditions. These results indicate that salt stress greatly reduced the number of DEGs between WT and *res* roots, whereas in leaves each condition stimulated specific subsets of genes, being slightly higher the number of DEGs in salt-stressed leaves. Moreover, we found that the majority of DEGs were tissue-specific (Fig. 2b). The reduction of DEGs in *res* vs WT roots under salt stress coincided with a significant difference in the magnitude of the transcriptomic response of each genotype in salt vs control (Additional file 2: Figure S2a). Thus, 1726 DEGs were found in salt-stressed *res* roots compared to non-stressed ones, and only 428 DEGs in WT. However, a high number of DEGs were detected in salt-treated vs control leaves of both genotypes (2793 in WT and 2169 in *res*), of which more than 50% were specifically altered in each genotype (Additional file 2: Figure S2a).

Functional classification of DEGs was carried out using the Mapman bin system [20]. The functional categories showing the highest number of DEGs between *res* and WT included genes with unknown function (around 15% of DEGs in each tissue and condition), followed by TFs, protein metabolism and signalling in both roots and leaves (Fig. 2c). Moreover, most of these functional categories coincided when considering DEGs in salt stress vs control for each genotype (Additional file 2: Figure S2b). The complete Mapman gene lists of relevant functional categories for each comparison (*res* vs WT in control and salt, as well as salt vs control for each genotype), are included in Additional files 3, 4, 5, 6, 7, 8 and 9: Tables S2-S8. Mapman diagrams showing that highly-altered functional groups were integrated within stress responses is presented in Additional file 10: Figure S3. In addition, selected DEGs with high up- and down-regulation are presented for each functional category and described in the following sections (Tables 1, 2, 3, 4, 5, 6, 7).

**Table 1:**
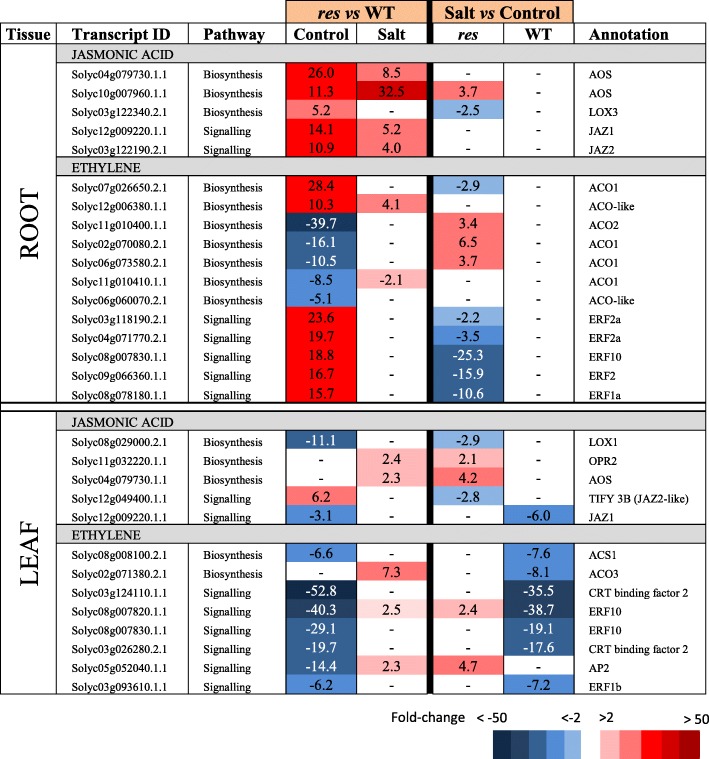
Genes involved in hormone metabolism showing high expression differences in roots and leaves of *res* and WT plants in absence of salt stress (Control) and after 5 days of 200 mM NaCl (Salt). Fold-change values are shown, comparing *res* vs WT in each condition (left columns) or salt stress vs control for each genotype (right columns). Fold-change values are also displayed by a colour scale, where blue represents down-regulation and red up-regulation in each given comparison

**Table 2:**
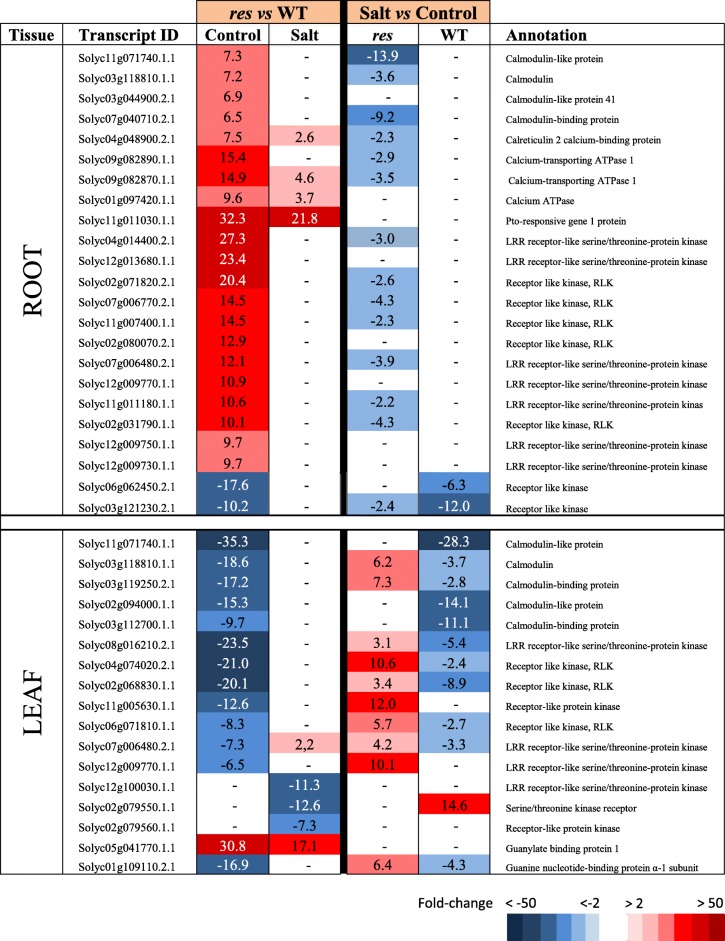
Selected genes involved in signalling pathways with high expression differences in roots and leaves of *res* and WT plants in absence of salt stress (Control) and after 5 days of 200 mM NaCl (Salt). Fold-change values are shown, comparing *res* vs WT in each condition (left columns) or salt stress vs control for each genotype (right columns). Fold-change values are also displayed by a colour scale, where blue represents down-regulation and red up-regulation in each given comparison

**Table 3:**
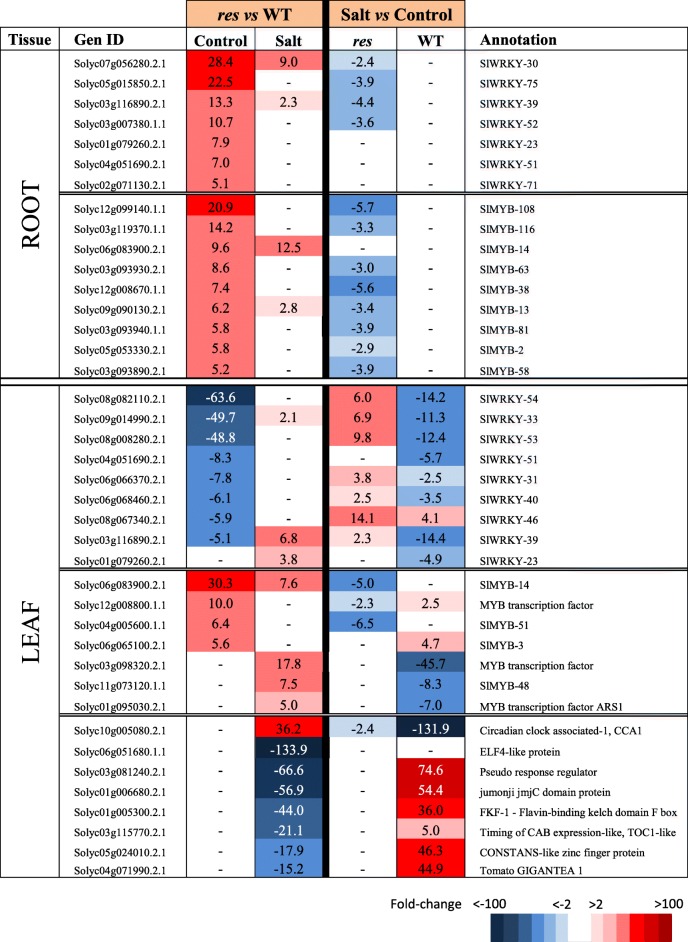
Selected DEGs encoding transcription factors showing high expression differences in roots and leaves of *res* and WT plants in absence of salt stress (Control) and after 5 days of 200 mM NaCl (Salt), considering the comparison between genotypes (*res* vs WT) for each treatment (left columns), and treatments (salt vs control) for each genotype (right columns). Colour panels and values display fold change values, where blue colour and negative data represent down-regulation, whereas red colour and positive data mean up-regulation

**Table 4:**
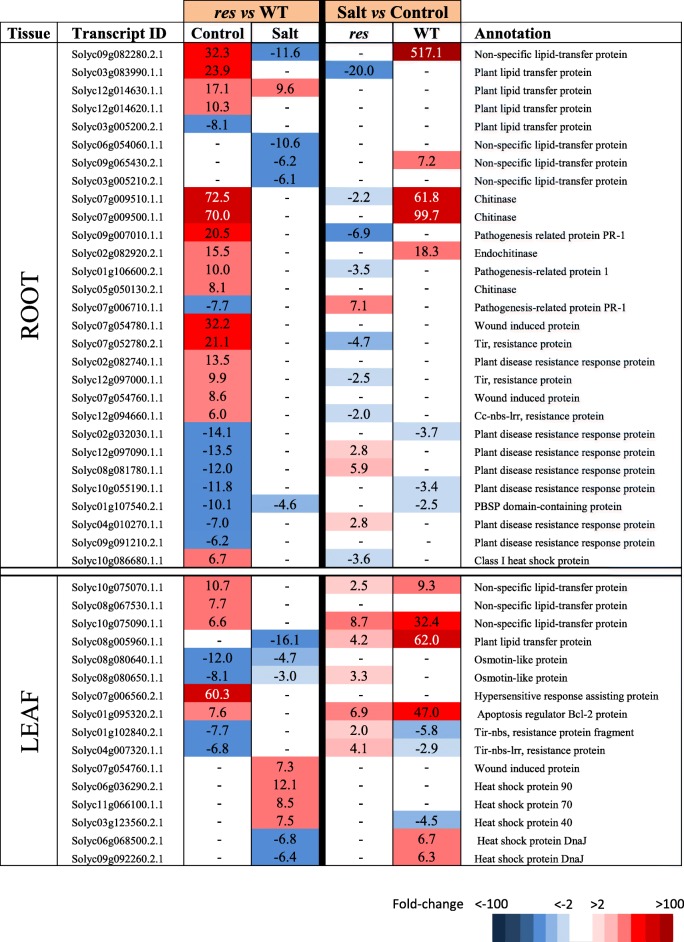
Selected DEGs encoding stress-related proteins showing high expression differences in roots and leaves of *res* and WT plants in absence of salt stress (Control) and after 5 days of 200 mM NaCl (Salt), considering the comparison between genotypes (*res* vs WT) for each treatment (left columns), and treatments (salt vs control) for each genotype (right columns). Colour panels and values display fold change values, where blue colour and negative data represent down-regulation, whereas red colour and positive data mean up-regulation

**Table 5:**
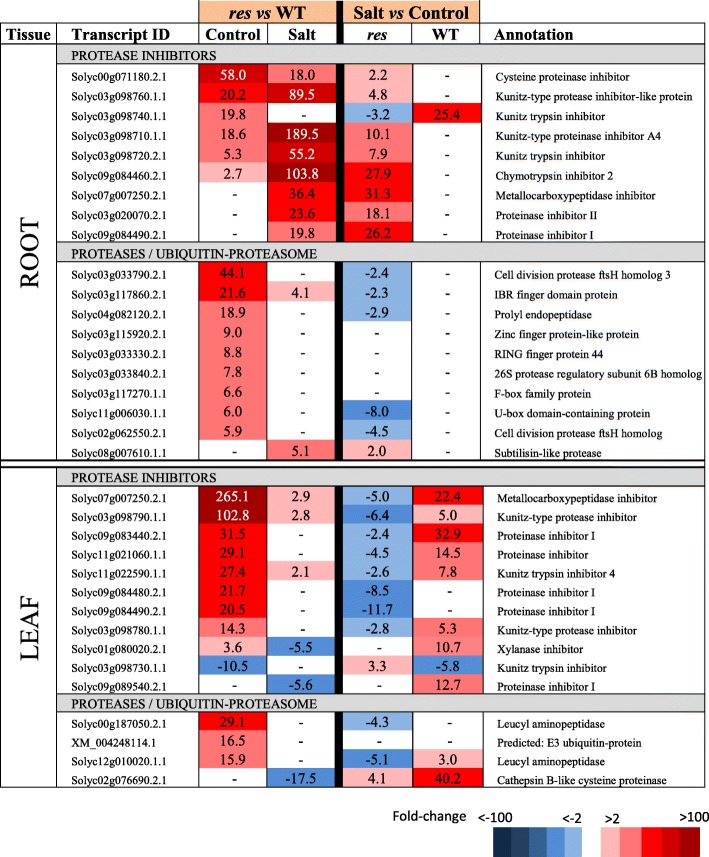
Selected DEGs involved in protein degradation showing high expression differences in roots and leaves of *res* and WT plants in absence of salt stress (Control) and after 5 days of 200 mM NaCl (Salt). Fold-change values are shown, comparing *res* vs WT in each condition (left columns) or salt stress vs control for each genotype (right columns). Fold-change values are also displayed by a colour scale, where blue represents down-regulation and red up-regulation in each given comparison

**Table 6:**
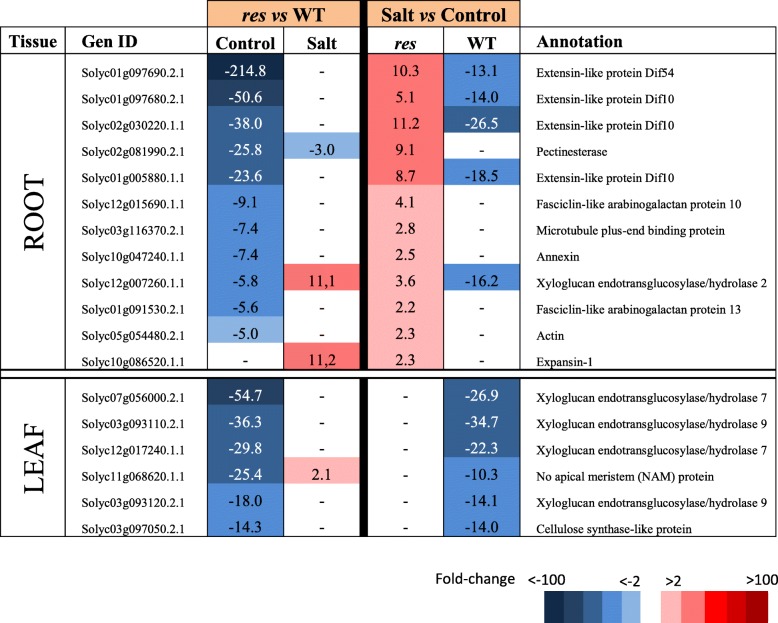
Selected DEGs involved in developmental processes, cell wall modification and cell cycle and organisation having high expression differences in roots and leaves of *res* and WT plants in absence of salt stress (Control) and after 5 days of 200 mM NaCl (Salt), considering the comparison between genotypes (*res* vs WT) for each treatment (left columns), and treatments (salt vs control) for each genotype (right columns). Colour panels and values display fold change values, where blue colour and negative data represent down-regulation, whereas red colour and positive data mean up-regulation

**Table 7:**
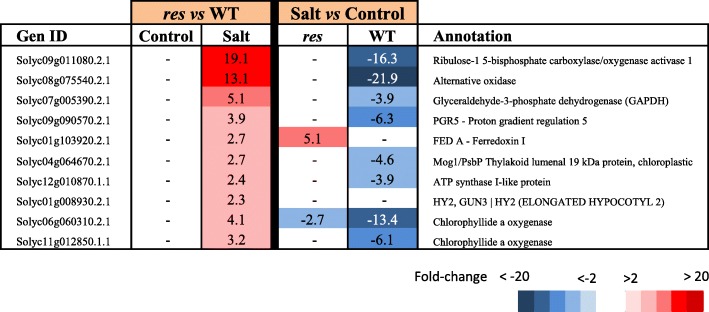
Photosynthesis-related genes with high expression differences in leaves of *res* and WT plants in absence of salt stress (Control) and after 5 days of 200 mM NaCl (Salt), considering the comparison between genotypes (*res* vs WT) for each treatment (left columns), and treatments (salt vs control) for each genotype (right columns). Colour panels and values display fold change values, where blue colour and negative data represent down-regulation, whereas red colour and positive data mean up-regulation

### Genes related to hormone metabolism and signalling pathways

One of the main differences in gene expression between *res* and WT was related to hormone metabolism (Additional file 3: Table S2), and concretely JA and ethylene (ET) metabolism genes showed the highest transcript differences between WT and *res* (Table 1). In roots, transcripts of genes related to JA biosynthesis (*AOS, LOX*) and signalling (*JAZ*) were significantly up-regulated in *res* vs WT, and expression differences were generally reduced in salt. Regarding ET, in roots no clear trend was observed for biosynthesis genes, whereas several *ETHYLENE RESPONSE FACTORS (ERFs*) genes were constitutively overexpressed. In leaf, however, most genes related to ET metabolism were down-regulated in control. Finally, in both tissues, most expression differences were reduced or disappeared in salt stress (Table 1).

The transcriptomic profiles of genes involved in signalling pathways differed significantly between roots and leaves in control plants (Additional file 4: Table S3), as most genes showing high expression differences were up-regulated in *res* roots and down-regulated in *res* leaves compared to WT (Table 2). Among the genes induced in non-stressed *res* roots we found a group of Ca^2+^-binding proteins such as calmodulins, a calreticulin, two Ca^2+^-transporting ATPases and a high number of receptor-like kinases. Contrarily, in leaves most of these genes were down-regulated in *res* compared to WT plants, with some exceptions like the gene encoding *GUANYLATE-BINDING PROTEIN 1*, classified by Mapman as putatively involved in G-protein signalling, which was highly up-regulated in *res* compared to WT leaves both in control and salt stress. The opposite response in roots and leaves was clearly shown by some transcripts found in both tissues. For example, Solyc03g118810.1.1 and Solyc11g071740.1.1, encoding a calmodulin and a calmodulin-like protein, respectively, and two LRR receptor-like serine/threonine protein kinases encoded by Solyc07g006480.2.1 and Solyc12g009770.1.1, were up-regulated in roots and repressed in leaves of *res* vs WT plants. Under salt stress, most gene expression differences between *res* and WT were significantly reduced in roots and leaves. However, whereas in roots this was mainly related to expression changes of *res* in salt vs control, in leaves it was due to changes induced by salt stress in either *res* or WT, or in both genotypes.

### Genes related to transcription factors and stress-related proteins

In control roots, the TFs up-regulated in *res* mutant included a numerous group of WRKYs and MYBs, while in salt stress most expression differences disappeared, in most cases due to their specific down-regulation in *res* roots under salinity (Additional file 5: Table S4, Table 3). In leaves, MYBs were also up-regulated in *res* vs WT, and some of them specifically under salt stress, such as *SlMYB48* and *ARS1*. On the other hand, DEGs between *res* and WT leaves encoding WRKY TFs showed opposite trends under control conditions compared to those observed in roots (for example *SlWRKY39*). In addition, a group of DEGs involved in circadian rhythm were specifically detected in leaves of *res* vs WT under salt stress, mainly related to the alteration of these genes in WT leaves upon salinity (Table 3). Interestingly, except the gene encoding MYB-like *CIRCADIAN CLOCK ASSOCIATED 1 (CCA1*), which was up-regulated, the rest were strongly down-regulated in *res* vs WT, including *TIMING OF CAB EXPRESSION1-LIKE (TOC1-LIKE*) and *GIGANTEA1*.

Regarding stress-related DEGs, several lipid transfer proteins (LTPs) were detected in *res* roots and leaves before any stress was applied, and these genes were generally up-regulated in both plant organs (Additional file 6: Table S5 and Table 4). Moreover, chitinases and pathogenesis-related proteins (PRs) were among the DEGs constitutively overexpressed in control, while other genes associated to biotic stress were down-regulated in *res* roots. In leaves, there was an increase in the number of DEGs from stress-related proteins in salt compared to control (Fig. 2c), and this included the specific up-regulation of several *HEAT SHOCK PROTEIN* (*HSPs)* genes in *res* compared to WT (Table 4).

### Genes involved in protein metabolism

In absence of stress, a high number of genes involved in protein degradation (proteases) and its regulation (protease inhibitors, PIs), including components of the ubiquitin-proteasome complex, were overexpressed in *res* roots and leaves compared to WT, with the highest expression differences corresponding to protease inhibitors (Additional file 7: Table S6, Table 5). While DEGs of proteases and ubiquitin-proteasome were mostly reduced in roots upon salt stress, several PIs showed higher overexpression values in salt than in control, especially the *KUNITZ-TYPE PROTEINASE INHIBITOR A4*. Moreover, other PIs were specifically up-regulated in *res* roots during salt stress, i.e. the gene *METALLOCARBOXYPEPTIDASE INHIBITOR (MPI)* (Solyc07g007250.2.1). In fact, this gene showed the highest up-regulation in *res* leaves in control conditions. It is also interesting to point out that several protease inhibitors detected are encoded by adjacent loci in the same chromosome (Solyc03g098710*,* Solyc03g098720, etc.).

### Genes involved in plant development and photosynthesis

An important number of genes involved in developmental processes, cell wall modification and cell cycle and organisation were down-regulated in *res* roots and leaves compared to WT in control conditions (Additional file 8: Table S7, Table 6), which agrees with the important development alterations shown by the *res* mutant in non-stressful conditions. However, gene expression differences disappeared in salt stress, coinciding with the phenotypic normalisation shown by mutant plants, and this was frequently related to up-regulation of genes previously repressed in *res* roots, although in some cases it was also due to gene repression induced by salt stress in WT.

In *res* leaves, significant differences in the expression of genes involved in photosynthesis and other related metabolic processes were found specifically in salt stress (Additional file 9: Table S8, Table 7). Among them, several components of the photosynthetic protein complexes, such as the *PsbP* subunit of PSII, *PGR5, FEDA* and *ATP SYNTHASE I-LIKE PROTEIN*, as well as two genes encoding CHLOROPHYLLIDE A OXYGENASES responsible of chlorophyll b synthesis. Interestingly, two genes that affect photosynthetic efficiency, *RUBISCO ACTIVASE 1 (RCA1)* (Solyc09g011080.2.1) and *ALTERNATIVE OXIDASE 1A* (*AOX1A*) (Solyc08g075540.2.1), showed the highest fold-change values in salt-stressed leaves of *res* compared to WT. In most cases, the differences found between *res* and WT were due to the specific down-regulation of these genes in WT leaves under salt stress.

### Analysis by RT-qPCR of selected genes (validation of selected DEGs by RT-qPCR)

**Fig. 3:**
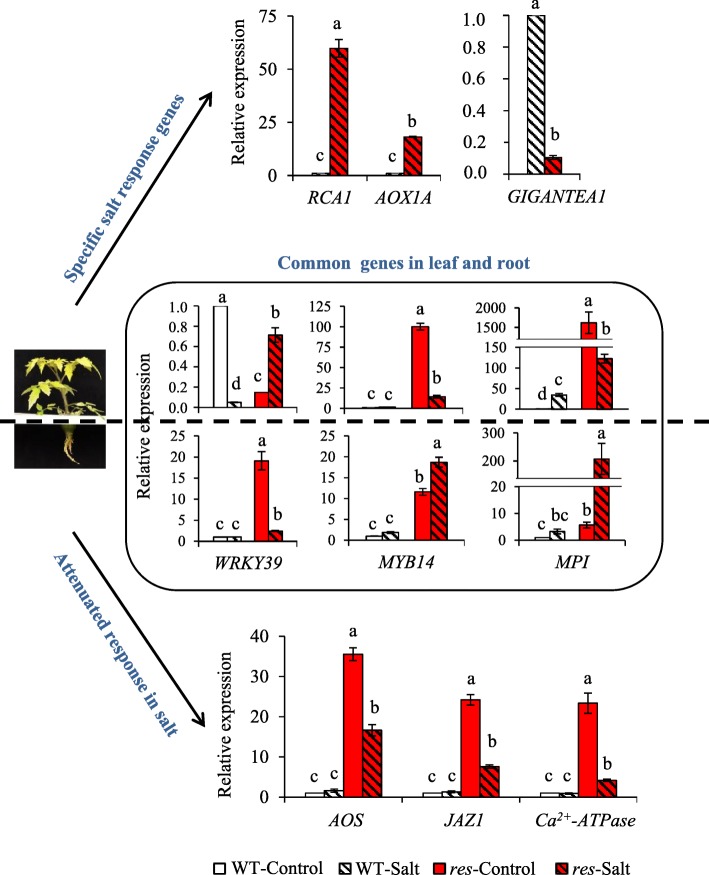
RT-qPCR analysis of relevant DEGs between WT and *res* in absence of salt stress (Control) and after 5 days at 200 mM NaCl (Salt). Specific salt response genes were analyzed in leaf (*GIGANTEA*, *RCA1* and *AOX1A*), common genes in both plant parts (*SlWRKY39, SlMYB14* and *MPI*), and genes of JA and Ca^2+^ signaling (*AOS, JAZ1* and Ca^2+^-ATPase) in roots. Values are means ± SE of three biological replicates. Different letters indicate significant differences between means by LSD test (*P < 0.05*)

Selected genes which seem to have a relevant role in the growth recovery and salt tolerance shown by the *res* mutant under salt stress were analysed by RT-qPCR. Among important DEGs in root, we tested two genes involved in JA metabolism (*AOS* and *JAZ1*) and one of signalling (*Ca*^*2+*^*-ATPase*), and found that salt stress reduced significantly the expression level of these genes in *res* roots compared to the level obtained in control (Fig. 3), thus corroborating that the constitutive alteration of these genes was attenuated by salt stress. In leaf, the two genes involved in photosynthetic efficiency were analysed, *RCA1* and *AOX1A*, and the data confirmed the great up-regulation of these genes in *res* vs WT under salt stress (Fig. 3). Contrarily, the circadian clock component *GIGANTEA1* was strongly repressed in *res* leaves compared to WT under salt stress. There were very few genes with differential expression in both tissues and conditions (14 DEGs), as shown in Fig. 2b, and interestingly two TFs, *WRKY39* and *MYB14,* were included among them. By RT-qPCR analysis (Fig. 3), we observed that *WRKY39* showed in root a similar response to that previously observed in JA genes, that is up-regulation in *res* vs WT in control, but important attenuation in salt stress. However, the expression of *MYB14* was not only higher in control but also in salt stress. In leaves, the expression changes of both genes showed opposite tendencies to those observed in roots, as *WRKY39* expression increased in *res* salt vs control, whereas the constitutive high expression of *MYB14* found in control leaves of *res* was strongly reduced by salt stress (86%). The other gene analysed was *MPI,* and the data corroborated that this PI was highly up-regulated in leaves, especially in control, and also in *res* roots during salt stress (Fig. 3).

In order to validate the microarray data, additional DEGs were analysed by RT-qPCR in roots and leaves (Additional file 11: Figure S4). Comparison of microarray and RT-qPCR results showed the same trend in relative gene expression, and a Pearson’s correlation coefficient of 0.87 was obtained (Additional file 11: Figure S4). In summary, the RT-qPCR data verified the reproducibility and reliability of the microarray analysis.

## Discussion

### The phenotype of *res* mutant is largely associated to the high constitutive expression of genes involved in stress responses and repression of genes related to growth and development in roots

The phenotype of *res* mutant comprises severe morphological and physiological dysfunctions and a high accumulation of the stress hormone JA in roots in absence of stress, while these alterations are suppressed by salinity [13]. A first question was to elucidate whether the development alterations in the *res* mutant were due to the high constitutive expression of stress genes. The comparative transcriptomic analysis between WT and *res* strongly supports that constitutive alterations in gene expression in roots account in great part for the phenotypical disorders of *res* mutant, as in control, the number of DEGs in *res* vs WT was 3-fold higher in roots than in leaves (3046 and 1019 DEGs, respectively) (Fig. 2a).

The root has a key role in abiotic stress such as salinity, as it is the first organ that enters in contact with salt and, consequently, its efficiency in stress detection and response mechanisms is determinant for stress tolerance [2, 3]. In this sense, the anticipated stress response of *res* roots before application of salinity, and the high number of altered genes in salt-stressed vs non-stressed *res* roots differs from the discrete response observed in WT roots upon salt stress (Additional file 2: Figure S2a). Among these, we hardly found DEGs in WT roots belonging to main functional categories, in contrast to *res* roots where numerous DEGs were detected (Tables 1–5). Thus, the transcriptomic analysis showed a constitutive enhanced expression of genes involved in JA biosynthesis (*AOS, LOX3*) and signalling (*JAZ*) in *res* roots (Table 1), which agrees with our previous results in JA accumulation [13]. Different studies indicate that JA and ET signalling often operate synergistically to activate mechanisms of abiotic stress tolerance [21], which also seems to occur in *res* roots, as several genes encoding *ERFs* were constitutively up-regulated in *res* roots in control conditions (Table 1). Interestingly, we also observed the up-regulation in *res* roots of numerous Ca^2+^ signalling genes (Table 2), which steer a multitude of downstream responses, as recently exemplified by studies on the function of Ca^2+^-ATPase [22]. The Ca^2+^ signalling aspects are beginning to be understood but still there is a great lack of information [23].

In agreement with the fact that hormones act as central integrators of stress adaptation signalling cascades, coordinating the transcriptional regulation of TFs and downstream genes [7], we also observed the up-regulation in *res* roots of TFs such as WRKYs and MYBs (Table 3), and a relatively high abundance of genes encoding stress-related proteins (Table 4), as lipid transfer proteins, which are involved in key physiological processes [24]. It is also interesting to mention that some of the genes encoding stress-related proteins identified are commonly associated with biotic stresses (Table 4), which agrees with our previous suggestions on the tolerance of the *res* mutant to multiple abiotic and biotic stresses [15].

On the other hand, growth-promoting genes were repressed in *res* vs WT roots in absence of stress but not under stress condition (Table 6), which agrees with the phenotypic normalisation shown by *res* mutant under salinity. This response is contrary to the generalized behaviour of plants, and similar to that observed in some wild tomato species and other halophytes, showing high expression levels of stress-relevant genes in absence of stress, which are determinant to their tolerance, but it usually provokes slower plant growth due to the energetic cost [16, 25].

### The phenotype recovery induced by salt stress in *res* mutant is mainly related to the attenuated response in root and enhanced in leaves

We observed that transcriptomic differences between *res* and WT were very attenuated in roots in salt stress (the number of DEGs was reduced by 90% compared to control) (Fig. 2a). In addition, we found that expression differences of genes involved in JA and ET metabolism, signalling pathways, TFs and stress-related proteins were significantly reduced or disappeared in salt, which was mainly due to the down-regulation of these genes comparing *res* mutant in salt vs control (Tables 1–4). These results relate the normalisation of *res* phenotype with an attenuated stress response in roots. The allocation of resources towards higher growth in *res* roots under salt stress is also supported by the significant up-regulation of genes involved in developmental processes (i.e. extensins, expansins and other cell wall modification genes) in salt-treated *res* roots compared to control, while the opposite trend was observed in WT plants (Table 6). This might reflect the higher salt-sensitivity of WT compared to *res* observed in the long-term [13].

On the other hand, the number of DEGs between *res* and WT was almost five times higher in leaves than in roots under salt stress (1366 vs 295 DEGs) (Fig. 2a), being stress-related proteins one of the functional categories with higher increase of DEGs in salt vs control (Fig. 2c). Taking into account that many stress-responsive genes display circadian expression [26], it is interesting to point out that salt-specific expression differences were observed for a group of circadian clock genes when *res* leaves were compared to those of WT, which were mainly a consequence of high expression levels in WT in salt vs control (Table 3). In this sense, [27] indicated that slower circadian rhythms allow better adaptation of the cultivated tomato to the long summer days (usually characterised by stressful environmental conditions), and our results suggest that this may be occurring in the response of the *res* mutant to salinity. Thus, the two principal components of the central oscillator, *CCA1* and *TOC1*, where *CCA1* acts as a repressor of *TOC1* expression [28]*,* were identified in our study. Moreover, other key clock components, i.e. *ELF4*, *CO, FKF-1*, the *JmJC* TF and *GIGANTEA (GI)* were down-regulated in *res* leaves vs those of WT in salt (Table 3). What is more, it has been reported that *gi* mutants are markedly salt tolerant, by exhibiting continued growth under salinity [16], as observed in *res* mutant. This observation agrees with the fact that *GI* is considered a negative regulator of salt tolerance, and precisely *GI* expression was strongly induced by salinity in WT (Table 3).

Finally, it is convenient to take into account that a significant amount of the genes detected do not have a known function or have not been functionally annotated to a specific process involved in the stress response (Fig. 2c), and thus they may also have a role, yet unknown, in the growth phenotype and salt response of *res* mutant. While in *res* roots most genes with unknown function were identified in control, in leaves the number of unknown DEGs was higher in salt than in control (Fig. 2c).

### Genes encoding MYB and WRKY transcription factors and protein metabolism may play prominent roles in the phenotype normalisation and salt tolerance shown by *res* mutant under salt stress

Only 14 genes differentially expressed in roots and leaves independently of the treatment were identified in the microarray analysis, including *WRKY39* and *MYB14*. The importance of *WRKY* and *MYB* genes in abiotic stress tolerance is well known [29, 30], although their roles in the salt tolerance of tomato are still scarcely known [31, 32]. We recently identified the *ARS1* gene, the first MYB of type R1 identified in tomato, which is involved in salt tolerance by regulating stomatal closure during stress and avoiding the high transport of Na^+^ up to the leaves [33]. Interestingly, in our transcriptomic study, *ARS1* was specifically salt-upregulated in *res* leaves compared to WT. Also *SlMYB48* (Table 3), a tomato homologous of *OsMYB48–1* which is known to enhance drought and salinity tolerance in rice [34]. Moreover, the *Arabidopsis thaliana AtWRKY40*, which is homologous to the *SlWRKY39* identified in this work, is known to stimulate JA-signalling by supressing JAZ repressors [35]. Both *WRKY39* and *MYB14* genes seem to have a role in the phenotype of *res* and its normalisation under salt stress, according to their expression profiles in roots and leaves of the mutant compared to WT (Fig. 3). Moreover, both genes showed opposite expression trends in *res* roots and leaves upon salinity, which suggests that they are specifically regulated in each tissue. Finally, it is interesting to point out that *MYB14* is one of the few genes increasing its expression in *res* roots in salt stress compared to control (Fig. 3).

The maintenance of protein homeostasis, i.e. the balance between protein biosynthesis and degradation, is crucial for plant survival [36]. We found remarkable expression differences between WT and *res* plants, in both tissues and treatments, for genes related to protein metabolism, especially those encoding proteases and PIs (Table 5). PIs have been generally associated to biotic stress responses [37], although they also seem to play important roles in abiotic stress responses, as adaptation to stress requires the active balance between regulated proteolysis and inhibition of uncontrolled proteolysis [38]. The overexpression of a cysteine protease inhibitor from *Jatropha curcas* in transgenic *Nicotiana benthamiana* conferred increased salinity tolerance along with lower MDA contents [39]. As PIs were highly up-regulated in *res* leaves in both conditions (Table 5), this could explain the constitutive lower MDA levels found in *res* leaves (Fig. 1c). One outstanding gene is *MPI* (Solyc07g007250.2.1), which showed the highest up-regulation in *res* leaves compared to WT, especially in control but also in salt stress. Moreover, *MPI* was highly up-regulated in *res* roots during salt stress (Fig. 3). Together, these results suggest that *MPI*, and probably other *PIs* genes, may have a role in the salt tolerance of *res*.

### The specific salt-upregulation of key genes involved in photosynthesis explain the enhancement of photosynthetic efficiency and phenotype recovery in *res* mutant

Under salinity, *res* plants were able to restore a normal phenotype and cell structure, including that of chloroplasts [13]. This fact explained the remarkable recovery of the greenish colour in leaves under salt stress, which was reflected in the increase in leaf chlorophyll content (Fig. 1c). Furthermore, an important physiological sign of salt tolerance in *res* mutant was the increase of the photosynthetic efficiency during salinity (Fig. 1c). Interestingly, only in some cases of halophytes, an increase in the photosynthetic activity with salinity may occur [40]. The increased photosynthetic efficiency of *res* was accompanied by specific salt-upregulation of genes involved in photosynthesis in the mutant compared to WT. Remarkably, the DEG with the highest fold change in salt-treated *res* leaves respect to WT was *RCA1* (Table 7). One important problem for improving plant productivity is optimizing the efficiency of Rubisco, as it is an extremely inefficient enzyme and needs a conformational repair, which is mediated by RCA [41]. In this sense, efforts to increase crop yields by bioengineering Rubisco remain unsuccessful [42]. By RT-qPCR we confirmed that *RCA1* was strongly upregulated in *res* leaves under salt stress compared to WT (Fig. 3). These higher expression levels in *res* may favour Rubisco reparation and its efficiency under salt stress.

Another gene with high up-regulation in *res* leaves compared to WT specifically in salt stress was *AOX1A* (Table 7, Fig. 3). AOX is able to efficiently oxidize the excess of reducing equivalents coming from chloroplast via the malate valve [43, 44]. In agreement, one of the genes conforming the malate valve, the chloroplast-localized *GLYCERALDEHYDE-3-PHOSPHATE DEHYDROGENASE* (*GAPDH*) (Solyc07g005390.2.1), was also up-regulated in *res* leaves under salt stress. Moreover, different studies revealed the importance of AOX pathway in optimizing photosynthesis under abiotic stress [45–48]. Thus, Arabidopsis plants overexpressing *AOX* displayed a higher relative growth rate than WT plants under drought stress [49], and in breeding studies, *AOX* was suggested as a marker to bring about efficient cell reprogramming during growth and development under stressful conditions [50]. Finally, although to lower levels, genes involved in light-reactions of photosynthesis were also specifically overexpressed in *res* leaves compared to WT under salt stress (Table 7), as *FED-A* and *PGR5* involved in electron flow in PSI, and *PspB* gene coding for a key protein of the PSII reaction centre.

Remarkably, all the genes related to photosynthetic efficiency were down-regulated in leaves of salt-treated WT plants compared to non-stressed leaves, especially *RCA1* and *AOX1A* (Table 7). The down-regulation of these genes in WT, along with those involved in developmental processes (i.e. expansins, extensins) are indicative of detrimental stress effects in WT plants, although physiological symptoms were not visible yet after 5 days of treatment. However, the increased growth and salt tolerance of the *res* mutant compared to WT plants may be related with the higher expression in salinity of key genes involved in optimizing photosynthesis, especially *RCA1* and *AOX1A*.

## Conclusions

The *res* mutant shows a remarkable growth penalty in absence of stress according to the high basal expression, especially in roots, of numerous genes involved in stress responses, from hormone metabolism, signalling, transcription factors, to other stress-related genes. However, the constitutive up-regulation of stress responses in *res* mutant confers a benefit upon exposure to salt stress and favour its phenotypic recovery, which is accompanied, on the one hand, by the attenuation of gene expression differences in roots and, on the other hand, by the enhancement of gene expression differences in leaves. In this sense, the down-regulation of *GIGANTEA1*, which is considered a negative regulator of salt tolerance, and the higher expression of genes involved in photosynthesis optimization, especially *RCA1* and *AOX1A*, may explain the higher photosynthetic efficiency and phenotypic recovery of *res* under salt stress. Remarkably, there are few DEGs between *res* and WT detected simultaneously in roots and leaves independently of the treatment, and these include *SlWRKY39*, *SlMYB14* and *MPI*, which seem to have a prominent role in the phenotype recovery of the mutant and its salt tolerance. Overall, our results illustrate how genes with different primary functions may work synergistically and coordinately in order to ensure successful plant adaptation to changing environmental conditions. Finally, we hope this work will provide new valuable information in order to enhance future breeding programs for stress-tolerant tomato crops.

## Methods

### Plant material and salt stress treatment

Plants of the tomato *res* mutant and *Solanum lycopersicum* L. cv. Moneymaker (wild-type, WT), were used in this work. Seeds were germinated in darkness, in a 8:3 (*v*/v) mixture of peat:perlite, at 28 °C temperature and 90% of relative humidity (RH). After emergence, plants were grown in a controlled growth chamber with 16 h light/8 h darkness photoperiod, with light of a photosynthetic photon flux (400–700 nm) of 350 μmol m^− 2^ s^− 1^ at the plant level, provided by fluorescent tubes (Philips Master TL-D 58 W/840 REFLEX, Holland), and 25 °C and 50–60% of temperature and RH, respectively. Two-week old plantlets were transferred to hydroponics. The hydroponic system consisted in tanks of 50 L volume (219 × 20 × 17 cm) filled with half-strength (½) Hoagland nutritive solution [51], which was continuously aerated by means of a compressor (Puska N-150-150, with a 115 L min^− 1^ flow, 10 Kg cm-^2^ maximum pressure). The nutritive solution was controlled by monitoring pH and electrical conductivity (EC), renewing it at least once per week.

Salt stress was applied to six-week old WT and *res* plants, and consisted in ½ Hoagland plus 200 mM NaCl as previously described [13]. Two previous assays were carried out in order to select the time of salt exposure where the phenotype recovery of *res* mutant began to be visible, and a time period of 5 days of salt stress was selected (Additional file 1: Figure S1). To avoid osmotic shock, 100 mM NaCl was applied during the first day and then 200 mM NaCl until the end of the treatment. Physiological measurements and harvesting of leaves (1st fully-expanded) and roots for molecular analyses were carried out in non-treated plants (control) and in plants salt-stressed for 5 days.

### Physiological measurements

All physiological measurements were determined in two independent salt stress assays, including three biological replicates (of three plants each one) per genotype and treatment. Chlorophyll content was analyzed in the 1st fully-developed leaf of WT and *res* plants by means of a portable device SPAD-502 that measures chlorophyll fluorescence (Minolta, Kyoto, Japan). The Soil Plant Analysis Development (SPAD) units given by the equipment are correlated with the plant chlorophyll content [52]. Three measurements were taken in different areas of the leaf, and the average value was obtained. Chlorophyll fluorescence was also analyzed by means of a portable Chlorophyll Fluorometer (Opti-Sciences, Hudson, NH). This equipment obtains the maximal photochemical efficiency of photosystem II estimated as Fv / Fm = (Fm − F_0_) / Fm, where Fv / Fm is the ratio between the variable fluorescence and the maximal fluorescence. Fm is the maximal fluorescence intensity in leaves adapted to darkness during 30 min, induced by a far red light excitation source (3000 μmol m^− 2^ s^− 1^) during 0.8 s. F_0_ is the minimal fluorescence intensity due to the exposition of leaves to an actinic light source (400 μmolm^− 2^ s^− 1^) [53].

Malondialdehyde (MDA) was quantified in leaflets from first fully-expanded leaves, as indicative of lipid peroxidation using the thiobarbituric acid reactive substrates (TBARS) assay, using the protocol described in [54] with slight modifications. Briefly, 0.5 g of leaflet tissue was homogenized in 4 mL of 0,1% trichloroacetic acid (TCA) solution using a Polytron (Kinematica AG, Switzerland). The homogenate was centrifuged at 15000 g for 10 min and 0.5 mL of the supernatant obtained was added to 1.5 mL 0.5% thiobarbituric acid (TBA) in 20% TCA. The mixture was incubated at 90 °C in a shaking water bath for 30 min, and the reaction was stopped by placing the reaction tubes in an ice-water bath. The samples were then centrifuged at 1000 g for 5 min, and the absorbance of supernatant was read at 532 nm. The value for non-specific absorption at 600 nm was subtracted. The amount of MDA-TBA complex (red pigment) was calculated from the extinction coefficient 155 m/M cm. Results were expressed as nmol MDA produced per gram of fresh weight per hour (nmol MDA g^− 1^ h^− 1^).

Data were statistically analysed using the SPSS 19.0 software package by two-way ANOVA, with means separated by least significant difference (LSD) (*P* < 0.05). All data are given as mean ± standard error (SE). Significant differences between means are denoted by different letters.

### Microarray analysis and data deposition

Microarray hybridization was performed in the Molecular Biology Section from Servicio de Apoyo a la Investigación (Universidad de Murcia, Spain). RNA isolated from leaflets (first fully-expanded leaf) and roots coming from three individual pooled plants (biological replicates) was used in hybridization to one chip, resulting in total 24 chips (three biological replicates for each genotype, tissue and treatment). RNA extraction was performed with RNeasy Mini kit (Qiagen, Hilden, Germany) according to manufacturer’s instructions, and the amount and quality of the RNA checked by Bioanalyzer and spectrophotometrically by Nanodrop® ND-2000 spectrophotometer (ThermoScientific, Waltham, MA, USA). ss-cDNA was synthetized from 100 ng of each sample using the GeneChip WT PLUS Reagent kit (Affymetrix, Santa Clara, CA, USA), according to the protocol supplied by the manufacturer. After quality-checked by Nanodrop and Bioanalyzer, ss-cDNA targets were cleaned up, fragmented and terminal-labelled. Then, 3.5 μg of fragmented and biotinylated ss-cDNA were included in the hybridization mix, using the Hybridization, Wash and Stain kit (Affymetrix) according to manufacturer’s recommendations. The resulting preparations were hybridized to the GeneChip® Tomato Gene 1.1 ST Array Strip (Affymetrix) offering whole-transcriptome coverage with 26 unique probes for each transcript (a total of 67,795 sequences are interrogated). After scanning, microarrays data were processed using Affymetrix Expression Comand Console (Affymetrix) and all samples (24 samples) overcame the quality criteria for hybridization and labelling tests. Data analysis were then performed with RMA (Robust Multiarray Average) allowing that raw intensity values were background corrected, log_2_-transformed and then quantile normalized in order to obtain an individual intensity value for each probeset. Non-supervised Principal Components Analysis (PCA) and hierarchical clustering were performed and they showed that samples tend to separate according to genotype and condition. Partek Genomics Suite and Partek Pathways software (Partek Incorporated, St. Louis, USA) were used for obtaining the annotation of probesets and performing the statistical analysis. An ANOVA test was applied with a restrictive threshold at *p*-value ≤0.05, and multiple test corrections was performed using the False Discovery Rate (FDR) [55]. Genes with FDR < 0.05 and fold change (ratio value) of ≥2.0 when comparing genotypes in the same experimental condition, or comparing different experimental conditions for each genotype, were identified as differentially expressed genes (DEGs). For the functional study of DEGs, Mapman software was used [20]. The Slyc_ITAG2.3 mapping was loaded and used for this functional analysis. The statistical analysis followed was of Wilcoxon Rank Sum test with Benjamini-Hochberg correction.

The microarray data and related analysis information from this work were deposited in NCBI’s Gene Expression Omnibus [56] and are accessible through accession number GSE106149 (https://www.ncbi.nlm.nih.gov/geo/query/acc.cgi?acc=GSE106149).

### Quantitative real-time PCR analysis

The expression levels of 14 selected genes (Additional file 12: Table S1) were analysed by quantitative real-time PCR (RT-qPCR) in order to validate the microarray expression profiles. 5 μg of total RNA extracts from roots and leaves of WT and *res* plants coming from the microarray experiment were used for cDNA synthesis with the First Strand cDNA Synthesis Kit (ThermoScientific, Waltham, MA, USA). 1 μg of the cDNA sample was used for gene amplification using the SYBR Green PCR Master Mix (Applied Biosystems, Foster City, CA, USA). Amplification reactions were carried out in a CFX Connect™ Real-Time PCR System (Bio-Rad, Hercules, CA, USA). All primers used for quantitative RT-qPCR are listed in Additional file 12: Table S1. Serial dilutions of cDNA samples were used to make a standard curve in order to calculate the amplification efficiency of primers (Additional file 12: Table S1). The presence of a single peak in the melting temperature curve confirmed the specificity of RT-qPCR amplification. Relative expression data were calculated as described by [57] using the elongation factor 1훼 (EF1훼, Solyc06g005060) as house-keeping gene. The expression level was calculated using 2^-ΔΔCt^ method [58], considering the expression level from WT as the calibrator sample. Three independent biological replicates were considered, each one consisting in three plants.

## Additional files

Additional file 1: **Figure S1.** WT and *res* mutant plants grown in hydroponics. The upper image shows plants grown in absence of stress (control). The lower image shows plants exposed to salt stress (200 mM NaCl for 5 days), where the reversion of the *res* phenotype is evident. (PPTX 1579 kb)

Additional file 2: **Figure S2.** Gene expression differences in WT and *res* plants comparing salt stress (200 mM NaCl for 5 days) and control conditions. (**a**) Venn diagrams showing the number of differentially expressed genes (DEGs) in roots (left) and leaves (right) when comparing salt stress vs control in WT and *res* plants. Numbers in parentheses are the total number of DEGs for each genotype and tissue. DEGs were identified as having FDR < 0.05 and a minimum fold-change value of 2.0. (**b**) Ranking of functional categories representing most number of DEGs in each tissue, according to Mapman classification, both in WT and *res* plants. (PPTX 67 kb)

Additional file 3: **Table S2.** Mapman classification of DEGs involved in hormone metabolism in roots (sheet 1) and leaves (sheet 2) of *res* and WT plants in absence of salt stress (Control) and after 5 days of 200 mM NaCl (Salt). (XLSX 75 kb)

Additional file 4: **Table S3.** Mapman classification of DEGs involved in signalling in roots (sheet 1) and leaves (sheet 2) of *res* and WT plants in absence of salt stress (Control) and after 5 days of 200 mM NaCl (Salt). (XLSX 114 kb)

Additional file 5: **Table S4.** Mapman classification of DEGs encoding transcription factors in roots (sheet 1) and leaves (sheet 2) of *res* and WT plants in absence of salt stress (Control) and after 5 days of 200 mM NaCl (Salt). (XLSX 115 kb)

Additional file 6: **Table S5.** Mapman classification of DEGs encoding stress-related proteins in roots (sheet 1) and leaves (sheet 2) of *res* and WT plants in absence of salt stress (Control) and after 5 days of 200 mM NaCl (Salt). (XLSX 69 kb)

Additional file 7: **Table S6.** Mapman classification of DEGs involved in protein metabolism in roots (sheet 1) and leaves (sheet 2) of *res* and WT plants in absence of salt stress (Control) and after 5 days of 200 mM NaCl (Salt). (XLSX 79 kb)

Additional file 8: **Table S7.** Mapman classification of DEGs involved in developmental processes in roots (sheet 1) and leaves (sheet 2) of *res* and WT plants in absence of salt stress (Control) and after 5 days of 200 mM NaCl (Salt). (XLSX 120 kb)

Additional file 9: **Table S8.** Mapman classification of DEGs involved in photosynthesis and related processes in leaves of *res* and WT plants in absence of salt stress (Control) and after 5 days of 200 mM NaCl (Salt). (XLSX 33 kb)

Additional file 10: **Figure S3.** Mapman stress diagrams. Differentially-expressed genes (DEGs) between *res* and WT in control and salt-stressed roots and leaves (200 mM NaCl for 5 days) involved in stress responses. Positive fold change values (red) indicate up-regulation (minimum fold-chang of 2.0) in *res* mutant compared to WT in each condition, whereas negative fold change values (blue) indicate down-regulation (minimum fold-change of − 2.0). Each coloured square represents an individual DEG. (PPTX 1566 kb)

Additional file 11: **Figure S4.** (**a**) Selected genes for completing the validation of the microarray analysis, apart from those shown in Fig. 3, and relative expression values obtained by RT-qPCR using the ΔΔCt method, where RNA from either leaflet or root tissue of WT plants grown in control was used as calibrator sample. Values are means ± SE of three biological replicates. (**b**) Correlation analysis between microarray (x-axis) and RT-qPCR (y-axis) data. The relative expression values obtained by microarray were compared with those obtained by RT-qPCR, and the Pearson’s correlation coefficient (R) was obtained (*R* = 0.87, *n* = 39). (PPTX 77 kb)

Additional file 12: **Table S1**. List of primers used for quantitative real-time RT-PCR. (XLSX 12 kb)

## Abbreviations

AOX: ALTERNATIVE OXIDASE
cDNA: Complementary deoxyribonucleic acid
DEG: Differentially Expressed Gene
EC: Electrical Conductivity
ERF: ETHYLENE RESPONSE FACTOR
ET: Ethylene
FC: Fold-change
FDR: False Discovery Rate
GI: GIGANTEA
HSP: Heat Shock Protein
JA: Jasmonic Acid/Jasmonate
LSD: Least Significant Difference
LTP: Lipid Transfer Protein
MDA: Malondialdehyde
MPI: METALLOCARBOXYPEPTIDASE INHIBITOR
NaCl: Sodium chloride
PCA: Principal Component Analysis
PI: Protease Inhibitor
PR: Pathogenesis-related protein
RCA: RUBISCO ACTIVASE
res: Restored cell structure by salinity
RH: Relative Humidity
RMA: Robust Multiarray Average
RNA: Ribonucleic acid
RT-qPCR: Quantitative reverse transcription polymerase chain reaction
SE: Standard Error
SPAD: Soil Plant Analysis Development
ss-cDNA: Single-stranded cDNA
TBA: Thiobarbituric Acid
TBARS: Thiobarbituric Acid Reactive Substrates
TCA: Trichloroacetic Acid
TF: Transcription Factor
WT: Wild-Type
