# Supplementary figures and images for: Identification of key genes involved in the phenotypic alterations of res (restored cell structure by salinity) tomato mutant and its recovery induced by salt stress through transcriptomic analysis
Source: BMC Plant Biol. 2018 Oct 1;18:213. doi: 10.1186/s12870-018-1436-9 (PMC6167845; doi:10.1186/s12870-018-1436-9)

## Slide 1
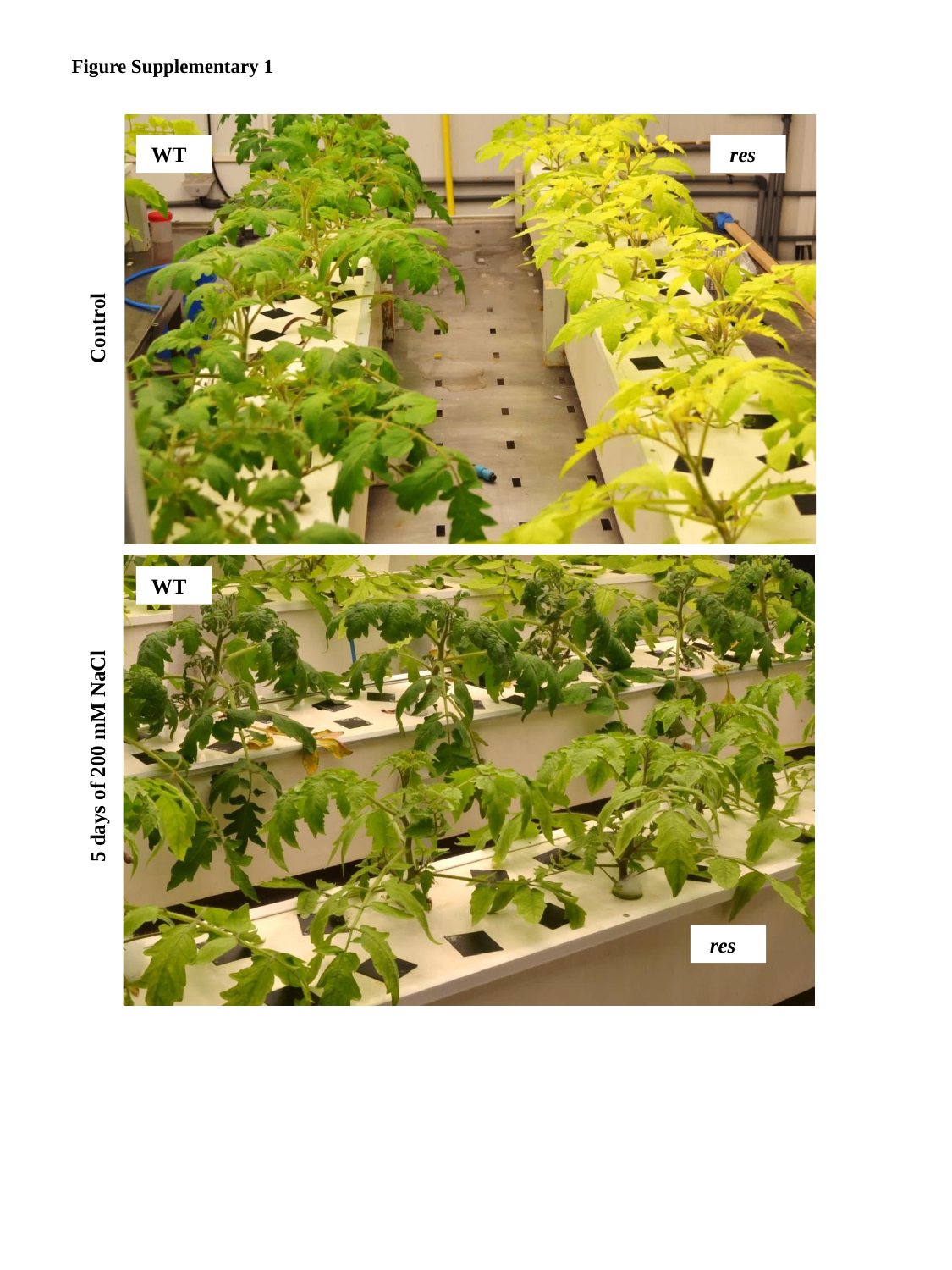

Figure Supplementary 1
WT
res
Control
5 days of 200 mM NaCl
res
WT

Supplement: Supplementary file 1 — Figure S1. WT and res mutant plants grown in hydroponics. The upper image shows plants grown in absence of stress (control). The lower image shows plants exposed to salt stress (200 mM NaCl for 5 days), where the reversion of the res phenotype is evident. (PPTX 1579 kb) [file 12870_2018_1436_MOESM1_ESM.pptx]

## Slide 1
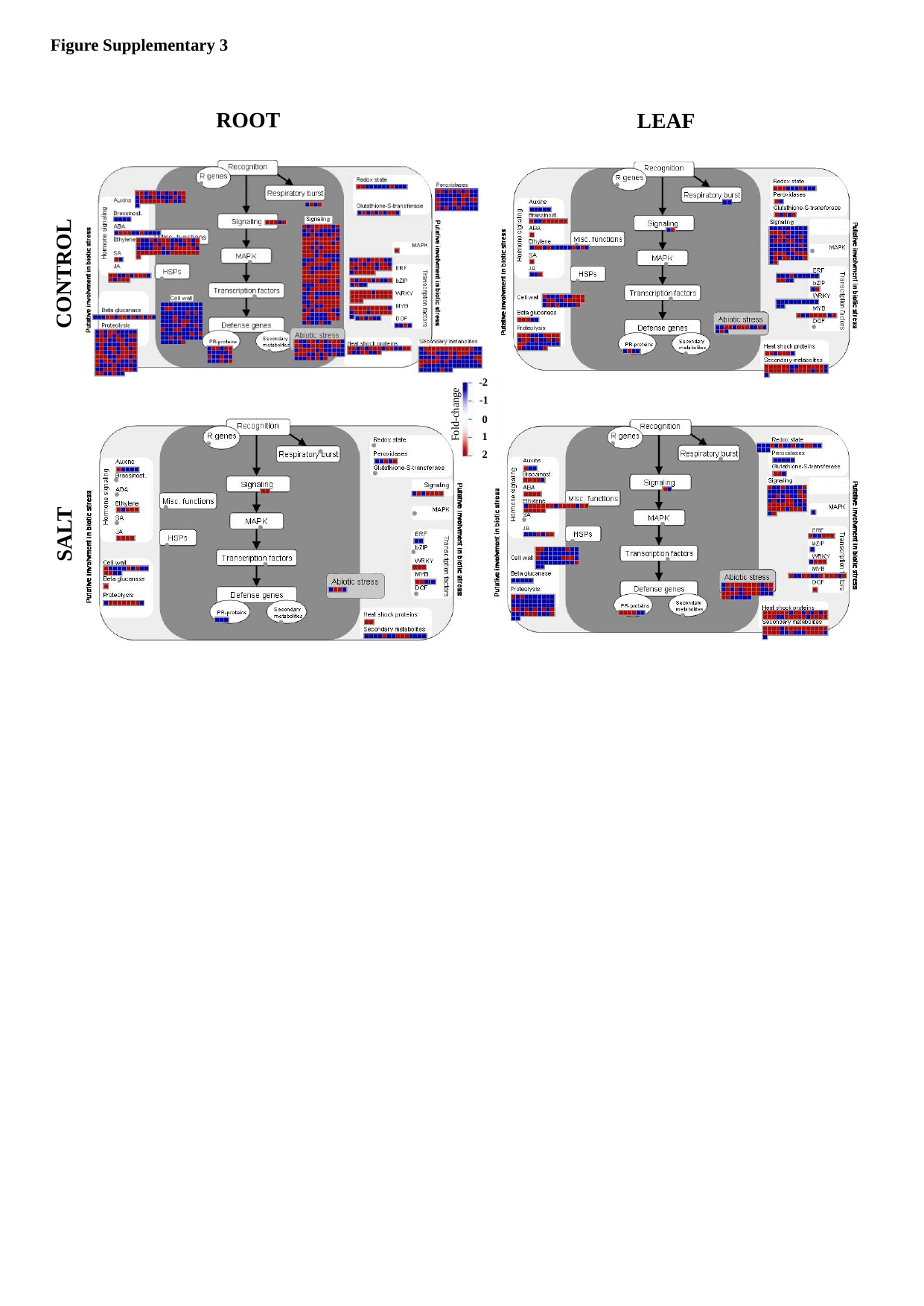

Figure Supplementary 3
ROOT
LEAF
CONTROL
-2
SALT
-1
Fold-change
 0
 1
 2

Supplement: Supplementary file 10 — Figure S3. Mapman stress diagrams. Differentially-expressed genes (DEGs) between res and WT in control and salt-stressed roots and leaves (200 mM NaCl for 5 days) involved in stress responses. Positive fold change values (red) indicate up-regulation (minimum fold-chang of 2.0) in res mutant compared to WT in each condition, whereas negative fold change values (blue) indicate down-regulation (minimum fold-change of − 2.0). Each coloured square represents an individual DEG. (PPTX 1566 kb) [file 12870_2018_1436_MOESM10_ESM.pptx]
