# Supplementary material for: Identification of key genes involved in the phenotypic alterations of res (restored cell structure by salinity) tomato mutant and its recovery induced by salt stress through transcriptomic analysis
Source: BMC Plant Biol. 2018 Oct 1;18:213. doi: 10.1186/s12870-018-1436-9 (PMC6167845; doi:10.1186/s12870-018-1436-9)
Supplement: Supplementary file 2 — Figure S2. Gene expression differences in WT and res plants comparing salt stress (200 mM NaCl for 5 days) and control conditions. (a) Venn diagrams showing the number of differentially expressed genes (DEGs) in roots (left) and leaves (right) when comparing salt stress vs control in WT and res plants. Numbers in parentheses are the total number of DEGs for each genotype and tissue. DEGs were identified as having FDR < 0.05 and a minimum fold-change value of 2.0. (b) Ranking of functional categories representing most number of DEGs in each tissue, according to Mapman classification, both in WT and res plants. (PPTX 67 kb) [file 12870_2018_1436_MOESM2_ESM.pptx]

## Slide 1
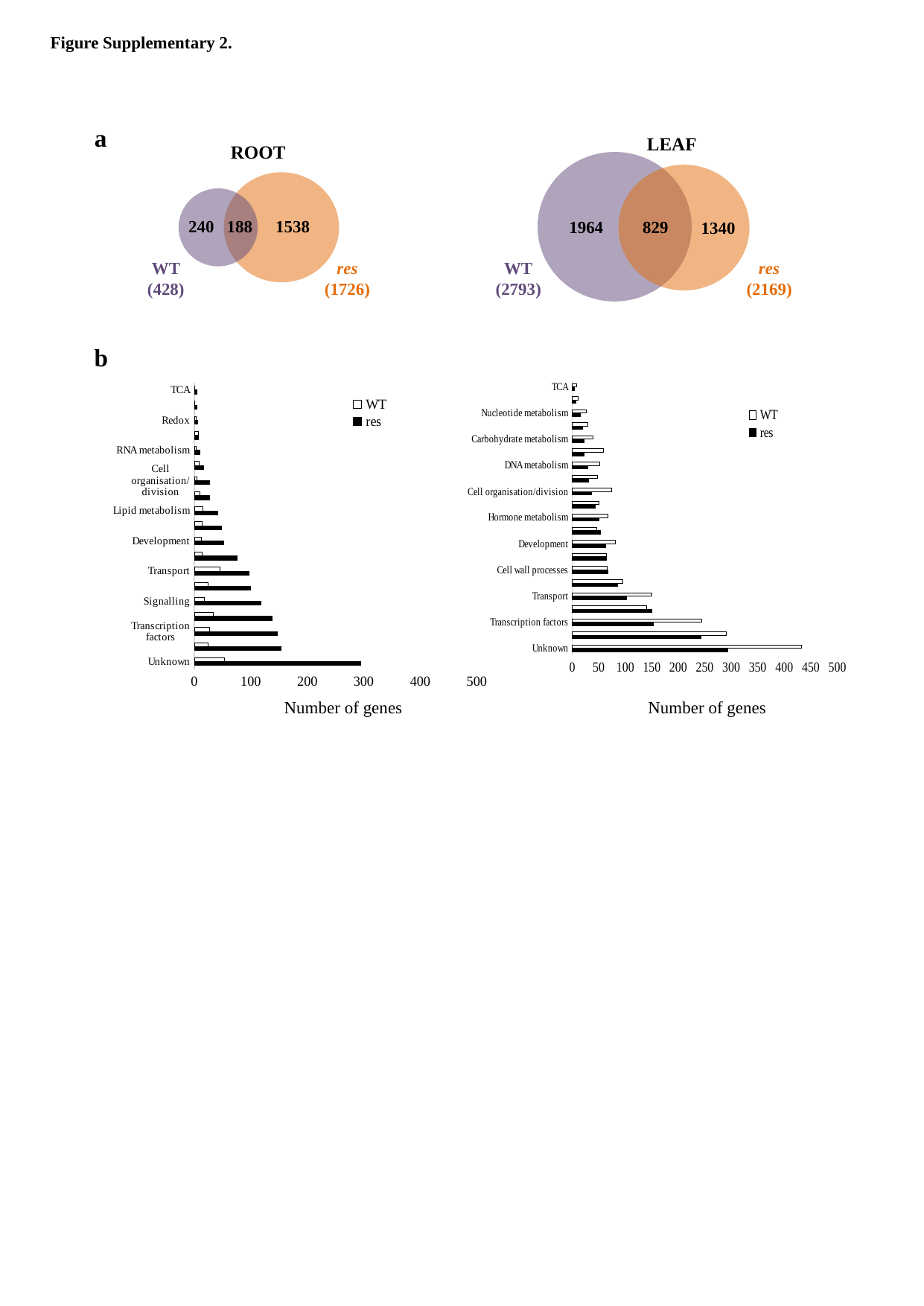

Figure Supplementary 2.
a
LEAF
ROOT
829
1964
1340
240
188
1538
WT
(428)
res
(1726)
WT
(2793)
res
(2169)
b
### Chart
| Category | | |
|---|---|---|
| Unknown | 295.0 | 54.0 |
| Cell wall processes | 153.0 | 24.0 |
| Transcription factors | 147.0 | 27.0 |
| Protein metabolism | 138.0 | 33.0 |
| Signalling | 118.0 | 18.0 |
| Stress-related proteins | 100.0 | 25.0 |
| Transport | 97.0 | 46.0 |
| Hormone metabolism | 76.0 | 14.0 |
| Development | 52.0 | 12.0 |
| Secondary metabolism | 48.0 | 14.0 |
| Lipid metabolism | 41.0 | 15.0 |
| Aminoacid metabolism | 27.0 | 10.0 |
| Cell organisation/division | 27.0 | 4.0 |
| Carbohydrate metabolism | 16.0 | 9.0 |
| RNA metabolism | 10.0 | 3.0 |
| DNA metabolism | 7.0 | 7.0 |
| Redox | 6.0 | 3.0 |
| Nucleotide metabolism | 5.0 | 1.0 |
| TCA | 4.0 | 1.0 |
### Chart
| Category | | |
|---|---|---|
| Unknown | 294.0 | 432.0 |
| Protein metabolism | 243.0 | 291.0 |
| Transcription factors | 153.0 | 245.0 |
| Signalling | 150.0 | 140.0 |
| Transport | 103.0 | 151.0 |
| Stress-related proteins | 86.0 | 95.0 |
| Cell wall processes | 67.0 | 66.0 |
| Lipid metabolism | 65.0 | 64.0 |
| Development | 63.0 | 82.0 |
| Secondary metabolism | 53.0 | 47.0 |
| Hormone metabolism | 50.0 | 68.0 |
| Aminoacid metabolism | 44.0 | 50.0 |
| Cell organisation/division | 37.0 | 75.0 |
| Photosynthesis | 31.0 | 48.0 |
| DNA metabolism | 30.0 | 52.0 |
| RNA metabolism | 23.0 | 59.0 |
| Carbohydrate metabolism | 22.0 | 40.0 |
| Redox | 19.0 | 29.0 |
| Nucleotide metabolism | 15.0 | 27.0 |
| Glycolysis | 7.0 | 11.0 |
| TCA | 5.0 | 8.0 |Number of genes
Number of genes
