# Supplementary material for: Identification of key genes involved in the phenotypic alterations of res (restored cell structure by salinity) tomato mutant and its recovery induced by salt stress through transcriptomic analysis
Source: BMC Plant Biol. 2018 Oct 1;18:213. doi: 10.1186/s12870-018-1436-9 (PMC6167845; doi:10.1186/s12870-018-1436-9)
Supplement: Supplementary file 11 — Figure S4. (a) Selected genes for completing the validation of the microarray analysis, apart from those shown in Fig. 3, and relative expression values obtained by RT-qPCR using the ΔΔCt method, where RNA from either leaflet or root tissue of WT plants grown in control was used as calibrator sample. Values are means ± SE of three biological replicates. (b) Correlation analysis between microarray (x-axis) and RT-qPCR (y-axis) data. The relative expression values obtained by microarray were compared with those obtained by RT-qPCR, and the Pearson’s correlation coefficient (R) was obtained (R = 0.87, n = 39). (PPTX 77 kb) [file 12870_2018_1436_MOESM11_ESM.pptx]

## Slide 1
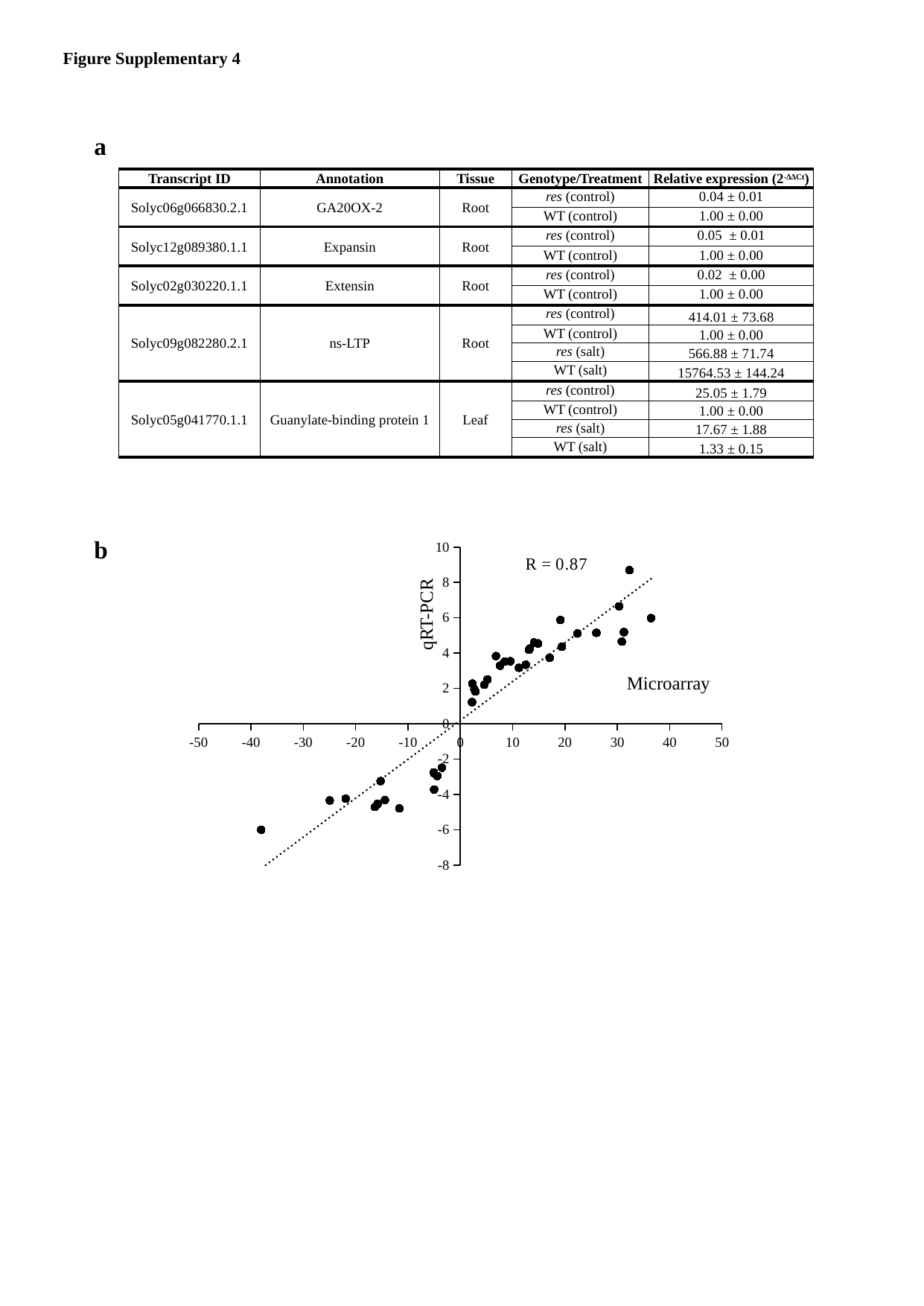

Figure Supplementary 4
a
| Transcript ID | Annotation | Tissue | Genotype/Treatment | Relative expression (2-∆∆Ct) |
| --- | --- | --- | --- | --- |
| Solyc06g066830.2.1 | GA20OX-2 | Root | res (control) | 0.04 ± 0.01 |
| | | | WT (control) | 1.00 ± 0.00 |
| Solyc12g089380.1.1 | Expansin | Root | res (control) | 0.05 ± 0.01 |
| | | | WT (control) | 1.00 ± 0.00 |
| Solyc02g030220.1.1 | Extensin | Root | res (control) | 0.02 ± 0.00 |
| | | | WT (control) | 1.00 ± 0.00 |
| Solyc09g082280.2.1 | ns-LTP | Root | res (control) | 414.01 ± 73.68 |
| | | | WT (control) | 1.00 ± 0.00 |
| | | | res (salt) | 566.88 ± 71.74 |
| | | | WT (salt) | 15764.53 ± 144.24 |
| Solyc05g041770.1.1 | Guanylate-binding protein 1 | Leaf | res (control) | 25.05 ± 1.79 |
| | | | WT (control) | 1.00 ± 0.00 |
| | | | res (salt) | 17.67 ± 1.88 |
| | | | WT (salt) | 1.33 ± 0.15 |
b
### Chart
| Category | |
|---|---|qRT-PCR
Microarray
